# Predicting the probability of pT3 or higher pathological stage at radical prostatectomy: COVID19-specific considerations

**DOI:** 10.3389/fonc.2022.990851

**Published:** 2022-12-06

**Authors:** Luigi Nocera, Lara F. Stolzenbach, Claudia Collà Ruvolo, Mike Wenzel, Christoph Wurnschimmel, Zhe Tian, Giorgio Gandaglia, Nicola Fossati, Vincenzo Mirone, Felix K. H. Chun, Shahrokh F. Shariat, Markus Graefen, Fred Saad, Francesco Montorsi, Alberto Briganti, Pierre I. Karakiewicz

**Affiliations:** ^1^ Cancer Prognostics and Health Outcomes Unit, Division of Urology, University of Montreal Health Center, Montreal, QC, Canada; ^2^ Division of Experimental Oncology/Unit of Urology, URI, Urological Research Institute, Istituto di Ricovero e Cura a Carattere Scientifico (IRCCS) San Raffaele Scientific Institute, Milan, Italy; ^3^ Martini-Klinik Prostate Cancer Center, University Hospital Hamburg-Eppendorf, Hamburg, Germany; ^4^ Department of Urology, University of Naples Federico II, Naples, Italy; ^5^ Department of Urology, University Hospital Frankfurt, Frankfurt am Main, Germany; ^6^ Department of Urology, Comprehensive Cancer Center, Medical University of Vienna, Vienna, Austria; ^7^ Departments of Urology, Weill Cornell Medical College, New York, NY, United States; ^8^ Department of Urology, University of Texas Southwestern, Dallas, TX, United States; ^9^ Department of Urology, Second Faculty of Medicine, Charles University, Prag, Czechia; ^10^ Institute for Urology and Reproductive Health, I.M. Sechenov First Moscow State Medical University, Moscow, Russia; ^11^ Division of Urology, Department of Special Surgery, Jordan University Hospital, The University of Jordan, Amman, Jordan

**Keywords:** radical prostatectomy, Pt3+, PT3, pT4, PCA, prostate cancer, C19

## Abstract

**Background:**

We tested whether a model identifying prostate cancer (PCa) patients at risk of pT3-4/pN1 can be developed for use during COVID19 pandemic, in order to guarantee appropriate treatment to patients harboring advanced disease patients without compromising sustainability of care delivery.

**Methods:**

Within the Surveillance, Epidemiology and End Results database 2010-2016, we identified 27,529 patients with localized PCa and treated with radical prostatectomy. A multivariable logistic regression model predicting presence of pT3-4/pN1 disease was fitted within a development cohort (n=13,977, 50.8%). Subsequently, external validation (n=13,552, 49.2%) and head-to-head comparison with NCCN risk group stratification was performed.

**Results:**

In model development, age, PSA, biopsy Gleason Grade Group (GGG) and percentage of positive biopsy cores were independent predictors of pT3-4/pN1 stage. In external validation, prediction of pT3-4/pN1 with novel nomogram was 74% accurate versus 68% for NCCN risk group stratification. Nomogram achieved better calibration and showed net-benefit over NCCN risk group stratification in decision curve analyses. The use of nomogram cut-off of 49% resulted in pT3-4/pN1 rate of 65%, instead of the average 35%.

**Conclusion:**

The newly developed, externally validated nomogram predicts presence of pT3-4/pN1 better than NCCN risk group stratification and allows to focus radical prostatectomy treatment on individuals at highest risk of pT3-4/pN1.

## Introduction

Extraordinary demands are placed on healthcare system during the COVID19 pandemic. In consequence, non-COVID19 related activities are curbed (1–3). Radical prostatectomies (RPs), regardless of risk level may be postponed for several months, according to most recent guidelines (4–13). This recommendation may be inconsistent with patients at particularly elevated risk of pT3-4/pN1 stage. Presence of pT3-4/pN1 stage may eliminate or substantially reduce the curative potential of deferred definitive therapy. In consequence, pT3-4/pN1 stage may not be compatible with radical prostatectomy deferment, even during COVID19 pandemic.

NCCN guidelines stratify prostate cancer (PCa) patients into very low, low, favorable intermediate, unfavorable intermediate, high and very high risk groups according to their clinical parameters, such as PSA levels, clinical T stage, Gleason Grade Group at biopsy, number and percentage of positive biopsy cores. The optimal therapeutic strategy differs among groups, ranging from active surveillance for very low and low risk patients, to surgery or radiotherapy for the other groups (14). The rates of pT3-4/pN1 in PCa patients are not negligible and range from 16% (favorable intermediate risk) to 60% (very high risk), depending on risk level (15–18). Due to highly variable rate of pT3-4/pN1 stage, even within risk level subsets, accurate identification of patients at particularly high pT3-4/pN1 risk is challenging. To date, a specific tool designed for prediction of pT3-4/pN1 stage in favorable intermediate, unfavorable intermediate, high risk or very high risk RP candidates has not been devised. We address this void. Specifically, we postulated that pT3-4/pN1 stage can be more accurately predicted than using the existing NCCN risk group stratification.

## Patients and methods

### Study population

In the Surveillance, Epidemiology and End Results (SEER) database (19), we focused on patients diagnosed with localized prostate cancer between 2010 and 2016, treated with radical prostatectomy (RP). Only individuals with known age, PSA level, clinical T stage, biopsy Gleason Grade Group (GGG), number of cores taken at biopsy (10 to 16), number of positive biopsy cores, pathological T stage and pathological N stage were included. Patients younger than 40 or older than 80 years were excluded, as well as patients with PSA level >40ng/ml (20). Patients harboring very low and low risk PCa were also excluded. Patients were then divided into two groups (development cohort and external validation cohort), based on region of residence. Specifically, the development cohort relied on patients from Midwestern, Southern and North-Eastern United States (n=13,977; 50.8%), according to SEER database. Conversely, the external validation cohort relied on patients from Western United States (n=13,552; 49.2%).

### Testing endpoint

The endpoint of interest consisted of the ability to identify patients with extra-prostatic disease at RP, defined as pT3-4/pN1, using clinical characteristics: age at diagnosis, PSA, clinical T stage, biopsy GGG and percentage of positive biopsy cores.

### Statistical analyses

Mean and standard deviations were reported for normally distributed, continuously coded variables. Median and interquartile ranges were reported for non-normally distributed, continuously coded variables. Frequencies and proportions were reported for categorical variables. The t-test, Mann-Whitney and chi-squared tests were used to compare means, medians and proportions, respectively. Statistical analyses consisted of several steps.

First, within the development cohort (n=13,977) we fitted a logistic regression model predicting pT3-4/pN1 using age at diagnosis, PSA level, biopsy GGG and percentage of positive biopsy cores. Clinical T stage was not included in the final model, due to lack of statistical significance. Moreover, based on left-skewed distribution of PSA level, cubic spline-transformed values were used in nomogram development.

Second, within the external validation cohort (n=13,552), we tested the discriminant ability of the newly developed nomogram, as well as that of the NCCN risk group stratification. Testing relied on ROC-derived area under the curve (AUC) that assessed the discriminant ability of nomogram versus the NCCN risk group stratification. Statistical significance of differences in related AUC values was tested according to DeLong et al. methodology (21). Moreover, comparisons of predicted versus observed probabilities of pT3-4/pN1 according to the nomogram, as well as according to the NCCN risk group stratification were depicted graphically in the form of calibration plots. Furthermore, decision curve analyses (DCA) tested the net-benefit related to the use of the nomogram versus the NCCN risk group stratification (22). Finally, systematic analyses of several possible nomogram cut-offs were performed.

All statistical tests were performed using the R statistical package v.3.6.1 (R Project for Statistical Computing, www.r-project.org) (23). All tests were two-sided, with a significance level set at p<0.05.

## Results

Within the SEER database, we identified 27,529 patients that harbored favorable intermediate, unfavorable intermediate, high risk or very high risk localized PCa, according to NCCN risk group stratification (14). Of those, 13,977 patients (50.8%) from Midwestern, Southern and North-Eastern United States formed the development cohort. The remaining 13,552 patients (49.2%) from Western United States formed the external validation cohort. Rate of pT3-4/pN1 was 18.3, 36.6, 42.5 and 66.7% in respectively favorable intermediate, unfavorable intermediate, high and very high risk PCa patients. Moreover, rate of pT3-4/pN1 was 32.5 and 35.5% in respectively development and external validation cohorts (p<0.001). Descriptive characteristics of the population and differences between the development and the external validation cohorts are depicted in **Table 1**.

**Table 1:** Descriptive characteristics of 19,193 patients with clinically localized prostate cancer treated with radical prostatectomy between 2010 and 2016, identified within SEER database.

Variable		Overall	Development cohort	External validation cohort	p value
		27,529 (100%)	13,977 (50.8%)	13,552 (49.2%)	
Age, yr	Mean (SD)	61.7 (0.042)	61.1 (0.058)	62.3 (0.059)	<0.001
	Median	62	61	63	<0.001
	IQR	57-67	56-66	58-67	
PSA, ng/ml	Mean (SD)	8.1 (0.034)	7.7 (0.046)	8.5 (0.05)	<0.001
	Median	6.3	6	6.7	<0.001
	IQR	4.8-9.6	4.6-9	5-10.1	
cT stage, n (%)	cT1	16858 (61.2)	8863 (63.4)	7995 (59.0)	<0.001
	cT2	10671 (38.8)	5114 (36.6)	5557 (41.0)	
GGG at biopsy, n (%)	1	3729 (13.5)	1766 (12.6)	1963 (14.5)	<0.001
	2	12903 (46.9)	6797 (48.6)	6106 (45.1)	
	3	5377 (19.5)	2744 (19.6)	2633 (19.4)	
	4	3554 (12.9)	1719 (12.3)	1835 (13.5)	
	5	1966 (7.1)	951 (6.8)	1015 (7.5)	
Percentage of positive biopsy cores, %	Mean (SD)	41.5 (0.147)	40.8 (0.203)	42.1 (0.214)	<0.001
	Median	38.5	37.5	40	<0.001
	IQR	23.1-58.3	23.1-57.1	23.1-58.3	
NCCN risk group, n (%)	Favorable intermediate	10662 (38.7)	5700 (40.8)	4962 (36.6)	<0.001
	Unfavorable intermediate	10487 (38.1)	5234 (37.4)	5253 (38.8)	
	High	2866 (10.4)	1321 (9.5)	1545 (11.4)	
	Very high	3514 (12.8)	1722 (12.3)	1792 (13.2)	
GGG at RP, n (%)	1	3392 (12.3)	1836 (13.1)	1556 (11.5)	<0.001
	2	13582 (49.3)	7044 (50.4)	6538 (48.2)	
	3	6218 (22.6)	2935 (21)	3283 (24.2)	
	4	1776 (6.5)	840 (6.0)	936 (6.9)	
	5	2261 (8.2)	1132 (8.1)	1129 (8.3)	
	Unknown	300 (1.1)	190 (1.4)	110 (0.8)	
pT stage, n (%)	pT2a	2029 (7.4)	1019 (7.3)	1010 (7.5)	<0.001
	pT2b	521 (1.9)	266 (1.9)	255 (1.9)	
	pT2c	14606 (53.1)	7414 (53.0)	7192 (53.1)	
	pT2x	1219 (4.4)	823 (5.9)	396 (2.9)	
	pT3x	9071 (33)	4403 (31.5)	4668 (34.4)	
	pT4	83 (0.3)	52 (0.4)	31 (0.2)	
pN stage, n (%)	0	18160 (66)	9084 (65.0)	9076 (67.0)	0.02
	1	1241 (4.5)	577 (4.1)	664 (4.9)	
pT3-4/pN1, n (%)	0	18172 (66)	9431 (67.5)	8741 (64.5)	<0.001
	1	9357 (34)	4546 (32.5)	4811 (35.5)	

GGG, Gleason Grade Group; RP, radical prostatectomy; IQR, interquartile range.

**Figure 1:**
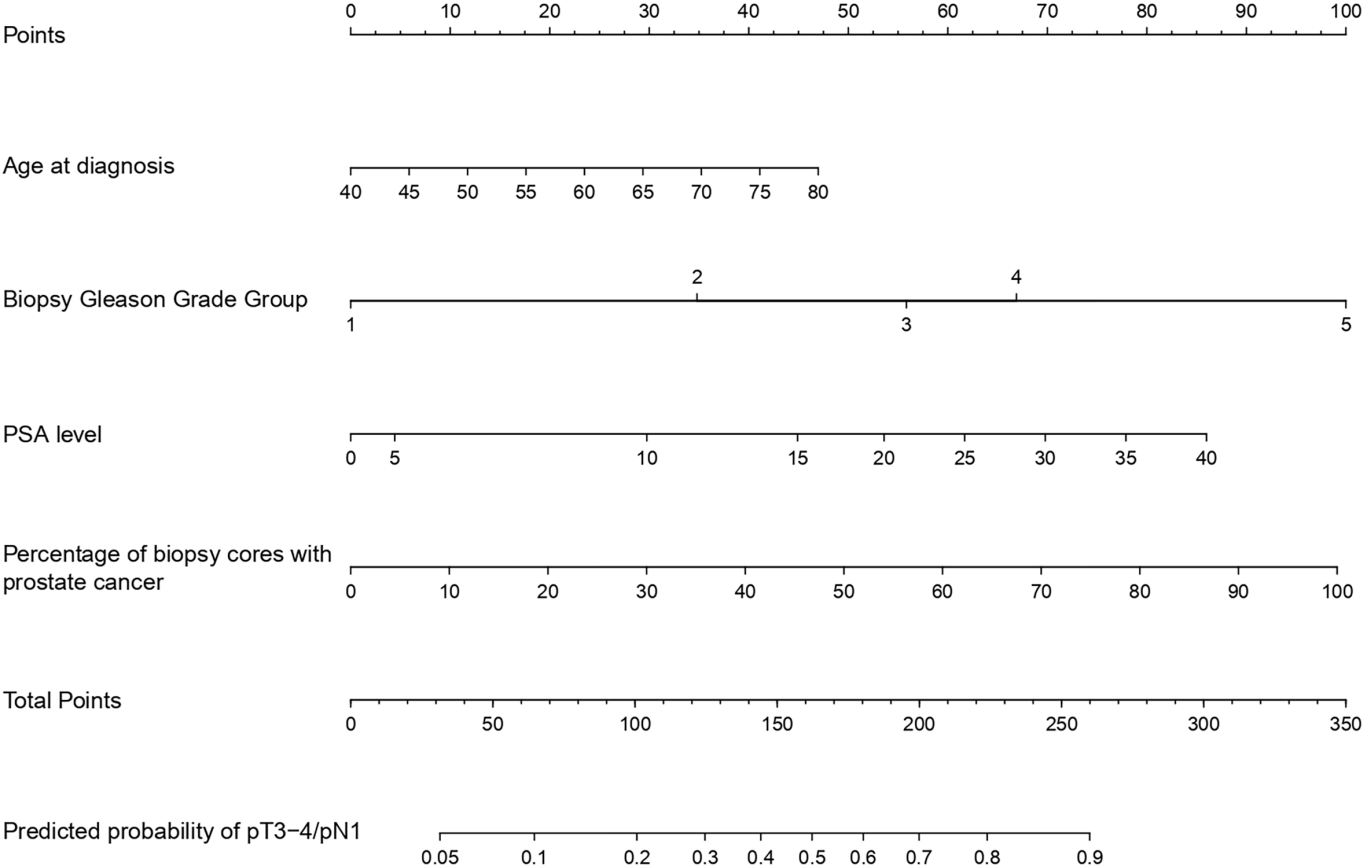
Model predicting the individual probability of pT3-4/pN1 at radical prostatectomy in prostate cancer patients.

Within the development cohort, the multivariable logistic regression model underlying the nomogram predicting the probability of pT3-4/pN1 rested on age, PSA level, biopsy GGG and percentage of positive biopsy cores (**Figure 1**). All included variable represented independent predictors. Within the nomogram, biopsy GGG and percentage of positive biopsy cores represented the two strongest contributors to total risk points and were followed by PSA level and age, in that order.

**Figure 2:**
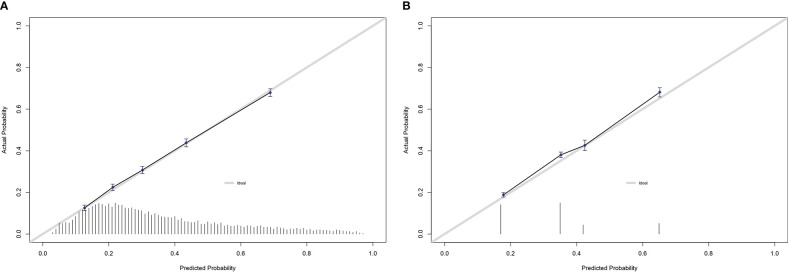
Calibration plots of observed versus predicted rates of pT3-4/pN1 within the external validation cohort of prostate cancer patients for: **(A)** the newly developed model; **(B)** NCCN risk group stratification.

**Figure 3:**
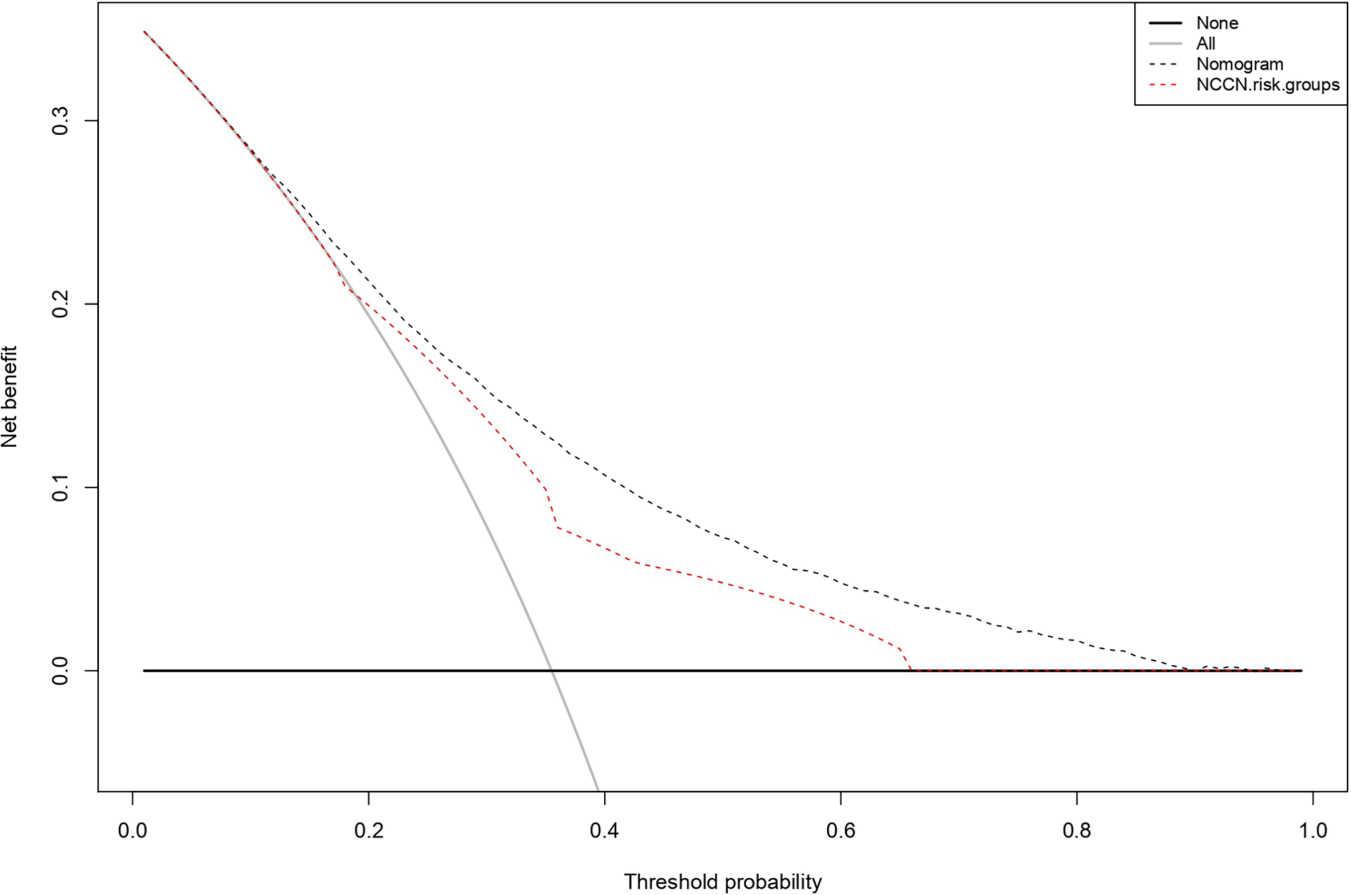
Decision curve analyses (DCA) demonstrating the net benefit associated with prediction of pT3-4/pN1 with the newly developed model versus a model based NCCN risk group stratification.

Within the external validation cohort, the nomogram yielded an AUC of 74.4% (95% CI 73.5 – 75.3%) versus 68.0% (95%CI 67.1 – 68.9%) for the NCCN risk group stratification (p<0.001). In calibration plots, comparisons between predicted and observed values yielded smaller departures from ideal predictions for the nomogram, relative to the NCCN risk group stratification. Specifically, for the nomogram departures from ideal predictions ranged from -0.9 to +1.2%, for five equally-sized groups. Conversely, for the NCCN risk groups departures from ideal predictions ranged from +0.1 to +2.9% (**Figure 2**). Moreover in DCA, greater degree of net-benefit was recorded for the nomogram, across all threshold probabilities, relative to the NCCN risk group stratification (**Figure 3**).

Finally, we tested specific nomogram cut-offs that corresponded to the predefined NCCN risk group levels. For example, a nomogram cut-off of 49% would identify 3,321 patients (24.5%) of the original 13,552 patients as at high risk of pT3-4/pN1. Of those individuals, 2,147 (64.6%) indeed harbored pT3-4/pN1. This cut-off virtually perfectly corresponded to the definition of high risk or higher, according to NCCN risk group stratification. This definition (high risk or very high risk) identified virtually the same number of individuals: 3,337 (24.6%) for NCCN versus 3,321 (24.5%) for the nomogram. Of those 3,337 individuals, 1,879 (56.3%) indeed harbored pT3-4/pN1, versus 2,147 out of 3,321 (64.6%) for the nomogram (**Table 2**). Alternatively, a more sensitive nomogram cut-off of 24% would identify 8,580 patients (63.3%) of the original 13,552 patients as at high risk of pT3-4/pN1. Of those individuals, 3,970 (46.3%) indeed harbored pT3-4/pN1. This cut-off virtually perfectly corresponded to the definition of unfavorable intermediate risk or higher, according to NCCN risk group stratification. This definition (unfavorable intermediate risk, high risk or very high risk) identified virtually the same number of individuals: 8,590 (63.4%) for NCCN versus 8,580 (63.3%) for the nomogram. Of those 8,590 individuals, 3,877 (45.1%) indeed harbored pT3-4/pN1, versus 3,970 out of 8,580 (46.3%) for the nomogram (**Table 2**). Finally, a more specific nomogram cut-off of 64% would identify 1,761 patients (13.0%) of the original 13,552 patients as at high risk of pT3-4/pN1. Of those individuals, 1,324 (75.2%) indeed harbored pT3-4/pN1. This cut-off virtually perfectly corresponded to the definition of very high risk, according to NCCN risk group stratification. This definition (very high risk) identified virtually the same number of individuals: 1,792 (13.2%) for NCCN versus 1,761 (13.0%) for the nomogram. Of those 1,792 individuals, 1,221 (68.1%) indeed harbored pT3-4/pN1, versus 1,324 out of 1,761 (75.2%) for the nomogram (**Table 2**).

**Table 2:** Rates of upstaging according to NCCN risk groups and nomogram cut-offs of 24%, 49% and 64%.

	Upstaging, n (%)	Upstaged to pT3-4, n (%)	Upstaged to pN1, n (%)
PCa patients within the external validation cohort 13,552 (100%)	4811 (35.5)	4699 (34.6)	664 (4.9)
NCCN unfavorable intermediate risk or higher PCa patients within the external validation cohort 8,590 (63.4%)	3877 (45.1)	3783 (44.0)	629 (7.3)
PCa patients above nomogram cut-off of 24% within the external validation cohort 8,580 (63.3%)	3970 (46.3)	3870 (45.0)	636 (7.4)
NCCN high risk or higher PCa patients within the external validation cohort 3,337 (24.6%)	1879 (56.3)	1822 (54.6)	413 (12.4)
PCa patients above nomogram cut-off of 49% within the external validation cohort 3,321 (24.5%)	2147 (64.6)	2091 (63.0)	486 (14.6)
NCCN very high risk PCa patients within external validation cohort 1,792 (13.2%)	1221 (68.1)	1188 (66.3)	304 (17.0)
PCa patients above nomogram cut-off of 64% within the external validation cohort 1,761 (13.0%)	1324 (75.2)	1287 (73.0)	361 (20.5)

## Discussion

During COVID19 pandemic RPs for localized intermediate or high risk PCa qualify for potential postponement up to 3 months or even beyond, according to guideline recommendations (4–13). However, some patients with clinically localized PCa may harbor pT3-4/pN1 disease. The latter can drastically curtail RP curative potential, especially if RP postponement of several months is applied. Currently, patients at elevated risk of pT3-4/pN1 cannot be identified with a specific clinical aid, except for NCCN risk group stratification. We hypothesized that a more accurate tool that allows dynamic interactions between multiple risk factors, instead of fixed NCCN risk group format, can be devised and that its ability to identify pT3-4/pN1 patients may exceed that of NCCN risk group stratification. Our analyses revealed several noteworthy observations.

First, within the overall cohort of 27,529 patients with clinically localized intermediate or high risk PCa, 34% of patients harbored pT3-4/pN1 stage. The rate of pT3-4/pN1 ranged from 18.3 to 66.7% in respectively favorable intermediate (18.3%), unfavorable intermediate (36.6%), high (42.5%) and very high (66.7%) risk PCa patients. According to individual patient characteristics, the rate of pT3-4/pN1 demonstrated important variability, even within those four risk groups. In consequence, the individual risk of pT3-4/pN1 stage is highly variable and cannot be precisely ascertained with the use of NCCN risk group stratification alone. In consequence, accurate prediction of individual pT3-4/pN1 probability prior to RP represents an unmet need. Previous nomograms developed by this group of investigators (24) and by others (25, 26) focused on RP patients of all risk levels. However, the majority RP candidates included in those reports harbored low risk PCa, and would not qualify for RP in 2020. Therefore, those previous reports do not qualify for consideration to develop COVID19-specific guideline recommendations for RP postponement. In consequence, the development of a new tool is needed and justified.

Second, to address the study objective, we relied on the development cohort of 13,977 patients residing in Midwestern, Southern and North-Eastern United States to identify independent predictors of pT3-4/pN1 at RP, within a multivariable logistic regression model. Those consisted of age, PSA level, biopsy GGG and percentage of positive biopsy cores. The logistic regression model was then graphically converted into nomogram format. Within that nomogram, biopsy GGG and percentage of positive biopsy cores contributed the highest possible number of risk points. Nonetheless, they were closely followed by PSA level and age. These observations indicate that patient, biochemical, as well as biopsy tumor characteristics represent important predictors of pT3-4/pN1 probability at RP. Moreover, in its graphical representation, the nomogram exhibits the importance of dynamic interactions between different levels of risk factors, regardless of their absolute values and across their entire range, without pre-defined cut-offs.

Third, the application of the newly developed nomogram in the external validation cohort yielded several important results. First, its accuracy was higher than the one of NCCN risk group stratification (74 vs 68%). Second, its calibration revealed lesser departures from ideal predictions than those of NCCN risk group stratification. Finally in DCA, a higher net-benefit was recorded for the nomogram than for the NCCN risk group stratification. Taken together, the newly developed nomogram exhibited better performance than the NCCN risk group stratification, according to three classic testing benchmarks for a predictive tool.

Fourth, although the nomogram can provide an individual probability of pT3-4/pN1, we relied on the use of cut-offs to illustrate its relative benefit versus NCCN risk group stratification. For example, the use of a 49% nomogram cut-off allowed us to identify 3,321 patients. Within those, 2,147 (64.6%) harbored pT3-4/pN1. The cut-off of 49% virtually perfectly replicated the NCCN definition of high risk PCa. The use of that definition also identified 3,337 patients. Of those, 1,879 (56.3%) harbored pT3-4/pN1. In consequence, despite virtually the same numbers of high risk individuals identified by the nomogram (3,321) versus NCCN risk group stratification (3,337), the rate of observed pT3-4/pN1 was higher for the nomogram than for the NCCN risk group stratification (64.6 vs 56.3%). In consequence, it may be concluded that the newly developed nomogram outperformed the NCCN risk group stratification based on three established statistical benchmarks. The use of nomogram cut-offs also yielded a higher proportion of individuals with pathologically confirmed pT3-4/pN1 than the NCCN risk group stratification. The same scenario was recorded when a lower nomogram cut-off (more sensitive) was compared to the NCCN risk group stratification, as well as when a higher nomogram cut-off (more specific) was compared to the NCCN risk group stratification.

Several nomograms have already been proposed for PCa patients stratification (17, 18, 27–30). However, they focused either on one specific risk group or on the totality of PCa patients independent of the risk group. Differently, our nomogram considered patients for whom active treatment in suggested, as per guidelines (14).

Taken together, we developed a new nomogram to identify individuals with pathologically proven pT3-4/pN1 stage, in contemporary favorable intermediate, unfavorable intermediate, high and very high risk patients. In those PCa patients, the nomogram outperformed the existing NCCN risk group stratification, based on the three established testing benchmarks, namely accuracy, calibration and DCA. Finally, the application of nomogram cut-offs resulted in a higher proportion of individuals with pathologically proven pT3-4/pN1 stage than the use of corresponding NCCN risk group definitions. In consequence, the use the nomogram represents a better alternative to NCCN risk group stratification.

Our study is not devoid of limitations, such as its retrospective and population-based nature that results in a limited number of assessable variables. For example, prostate magnetic resonance imaging findings, as well as molecular and genetic tests, repeat biopsy information or percentage of cancer per core were unavailable (31, 32). Moreover, SEER database does not allow adjustment or specific analyses that focus on later cancer control endpoints such as, for example BCR rates. Finally, lack of central review for biopsy, as well as RP pathology, also represents potential weakness (33, 34). However, it makes our findings generalizable to routine clinical practice, where central pathology is not available.

## Conclusion

The newly developed, externally validated nomogram predicts presence of pT3-4/pN1 better than NCCN risk group stratification and allows to focus radical prostatectomy treatment on individuals at highest risk of pT3-4/pN1, during COVID19 pandemic and similar crises.

## Data availability statement

The original contributions presented in the study are included in the article/supplementary material. Further inquiries can be directed to the corresponding author.

## Author contributions

LN: conception and design, acquisition of data, analysis and interpretation of data, drafting of the manuscript, critical revision of the manuscript, statistical analysis LS: acquisition of data, analysis and interpretation of data, critical revision of the manuscript CR: acquisition of data, analysis and interpretation of data, critical revision of the manuscript MW: acquisition of data, analysis and interpretation of data, critical revision of the manuscript CW: acquisition of data, analysis and interpretation of data, critical revision of the manuscript ZT: statistical analysis GG: critical revision of the manuscript NF: critical revision of the manuscript VM: critical revision of the manuscript FC: critical revision of the manuscript SS: critical revision of the manuscript MG: critical revision of the manuscript FS: critical revision of the manuscript FM: critical revision of the manuscript, supervision AB: critical revision of the manuscript, supervision PK: conception and design, drafting of the manuscript, critical revision of the manuscript, supervision. All authors contributed to the article and approved the submitted version.
